# mHealth to improve implementation of TB contact investigation: a case study from Uganda

**DOI:** 10.1186/s43058-023-00448-w

**Published:** 2023-06-20

**Authors:** Amanda J. Gupta, Patricia Turimumahoro, Emmanuel Ochom, Joseph M. Ggita, Diana Babirye, Irene Ayakaka, David Mark, Daniel Ayen Okello, Adithya Cattamanchi, David W. Dowdy, Jessica E. Haberer, Mari Armstrong-Hough, Achilles Katamba, J. Lucian Davis

**Affiliations:** 1 Department of Epidemiology of Microbial Diseases, Yale School of Public Health, New Haven, CT USA; 2 Uganda Tuberculosis Implementation Research Consortium, Makerere University, Kampala, Uganda; 3 Johns Hopkins Bloomberg School of Public Health, Baltimore, MD USA; 4 Kampala Capital City Authority, Kampala, Uganda; 5 Division of Pulmonary and Critical Care Medicine, University of California San Francisco, San Francisco, CA USA; 6 Division of Pulmonary Diseases and Critical Care Medicine, University of California, Irvine, Irvine, CA USA; 7 Department of Epidemiology, Johns Hopkins Bloomberg School of Public Health, Baltimore, MD USA; 8 Department of Medicine, Massachusetts General Hospital, Harvard Medical School, Boston, MA USA; 9 Department of Social and Behavioral Sciences, New York University School of Global Public Health, New York, NY USA; 10 Department of Epidemiology, New York University School of Global Public Health, New York, NY USA; 11 Clinical Epidemiology Unit, Department of Medicine, Makerere University, Kampala, Uganda; 12 Pulmonary, Critical Care, and Sleep Medicine Section, Yale School of Medicine, New Haven, CT USA; 13 Center for Methods in Implementation and Prevention Science, Yale School of Public Health, New Haven, CT USA

**Keywords:** Tuberculosis, Contact tracing, Africa, Digital technology

## Abstract

**Background:**

Implementation science offers a systematic approach to adapting innovations and delivery strategies to new contexts but has yet to be widely applied in low- and middle-income countries. The Fogarty Center for Global Health Studies is sponsoring a special series, “Global Implementation Science Case Studies,” to address this gap.

**Methods:**

We developed a case study for this series describing our approach and lessons learned while conducting a prospective, multi-modal study to design, implement, and evaluate an implementation strategy for TB contact investigation in Kampala, Uganda. The study included formative, evaluative, and summative phases that allowed us to develop and test an adapted contact investigation intervention involving home-based sample collection for TB and HIV testing. We concurrently developed a multi-component mHealth implementation strategy involving fingerprint scanning, electronic decision support, and automated reporting of test results via text message. We then conducted a household-randomized, hybrid implementation-effectiveness trial comparing the adapted intervention and implementation strategy to usual care. Our assessment included nested quantitative and qualitative studies to understand the strategy’s acceptability, appropriateness, feasibility, fidelity, and costs. Reflecting on this process with a multi-disciplinary team of implementing researchers and local public health partners, we provide commentary on the previously published studies and how the results influenced the adaptation of international TB contact investigation guidelines to fit the local context.

**Results:**

While the trial did not show improvements in contact investigation delivery or public health outcomes, our multi-modal evaluation strategy helped us identify which elements of home-based, mHealth-facilitated contact investigation were feasible, acceptable, and appropriate and which elements reduced its fidelity and sustainability, including high costs. We identified a need for better tools for measuring implementation that are simple, quantitative, and repeatable and for greater attention to ethical issues in implementation science.

**Conclusions:**

Overall, a theory-informed, community-engaged approach to implementation offered many learnings and actionable insights for delivering TB contact investigation and using implementation science in low-income countries. Future implementation trials, especially those incorporating mHealth strategies, should apply the learnings from this case study to enhance the rigor, equity, and impact of implementation research in global health settings.

Contributions to the literature

- Describe the use of community engagement and behavioral theory to adapt and implement an evidence-based public health intervention in a low-income country
- Explain the similarities and differences between adapting evidence-based interventions to local context and designing tailored implementation strategies
- Identify facilitators and barriers related to designing and evaluating mobile health strategies in a low-income country
- Present the strengths and weaknesses of different implementation measures and the opportunities to learn from negative trials through rigorous assessments of fidelity and context
- Review examples of specific ethical concerns that may arise during implementation trials and how they can influence study outcomes

## Background

In recent years, there has been a rapidly growing interest in using implementation science to tackle major health threats worldwide. Unfortunately, the uptake of implementation science has been less in low- and middle-income countries than in high-income countries [1], possibly reflecting more limited access to the necessary funding, infrastructure, human resources, and technical expertise to apply these methods. This “implementation gap in implementation science” presents unique challenges and opportunities for impact in the global health context [2]. To help address these gaps, the Center for Global Health at the Fogarty International Center is sponsoring a new collection of implementation science case studies. This collection aims to showcase rigorous approaches to conducting implementation research globally while highlighting practical considerations and challenges. As part of this series, we present a case study summarizing our experiences and lessons learned during the Mobile Health for Home TB Contact Investigation in Uganda study, a recently completed project described in several previous publications cited below.

The World Health Organization (WHO) has estimated that 10.6 million people developed active tuberculosis (TB) in 2021. After years of declining TB disease mortality, TB-related deaths rose to 1.6 million in 2021 [3], partly due to growing global poverty and decreased public health attention to TB during the COVID-19 pandemic. Contact investigation is an evidence-based public health intervention to advance TB control and elimination and consists of three core activities: (1) *enumerating* household and other close contacts of newly diagnosed TB patients; (2) *screening* them for possible active TB disease through a careful review of symptoms, demographic and clinical risk factors, and physical findings; and (3) *testing* them for TB infection and TB disease and linking them to preventative or curative treatment. Observational studies have long demonstrated that household contact investigation provides a substantial yield of new active and latent TB diagnoses [4] and is likely cost-effective [5]. More recently, high-quality randomized controlled trials have added comparative data showing that contact investigation effectively reduced active TB prevalence in one Brazilian community [6] and increased TB case notifications across 36 districts in Vietnam [7]. Three other randomized trials were inconclusive, with one strongly suggesting but unable to confirm a benefit of contact tracing over clinic-based case-finding [8] and two others showing no benefit over facility-based approaches [9, 10]. As a result, WHO now endorses TB contact investigation in all settings, including low- and middle-income countries (LMICs) [11, 12]. The heterogeneous results from more recent trials and routine implementation projects [13–17] all show the need for national TB policies [15] encouraging on implementing contact investigation to maximize impact.

Heterogeneity of intervention and implementation outcomes often occurs when interventions are adapted to fit the local context and resources. For example, in some settings, people diagnosed with TB may be responsible for notifying and referring their close contacts for TB screening (i.e., “contact invitation”); in others, health workers may telephone contacts to notify and refer them (i.e., “contact tracing”); and still elsewhere, health workers may visit and screen contacts in the community (i.e., “contact investigation”). In addition, algorithms for screening (i.e., no screening, symptom screening, chest radiography) and testing (i.e., sputum smear microscopy, GeneXpert MTB/RIF Ultra molecular testing) often vary between settings. Finally, programs may or may not offer TB preventive treatment to those who are exposed to TB or acquire TB infection but test negative for TB disease. Since home-based TB screening is the most common approach in Uganda, we will use the term “contact investigation” to characterize the intervention and its local adaptation.

Implementation strategies aim to eliminate heterogeneity of outcomes while allowing flexibility and adaptation to the local context. Mobile health (mHealth) technologies are popular and widely used to attempt to close gaps between guideline-recommended and routine practice [18–23]. Mobile devices, including telephones and tablets, can be deployed in the healthcare sector to achieve various implementation objectives, such as facilitating communication between patients and providers, improving data collection, and guiding recommendations for evaluation and treatment [24]. Nevertheless, there is relatively little empirical evidence that mHealth strategies are effective, especially in real-world settings in LMICs [25, 26]. There are even fewer studies describing generalizable mHealth strategies to improve implementation outcomes. As a result, most mHealth projects have yet to progress beyond the pilot phase [27]. One possible explanation is that few mHealth strategies incorporate robust behavioral components based on a well-articulated theory of change, a factor associated with failed implementation in other settings [28, 29]. There is some evidence that mHealth interventions that integrate behavioral theory may be more successful [30, 31] than those that do not, although it is unknown if these impacts are sustainable [32], especially as technologies evolve [33].

Uganda, a country with a high HIV and TB burden, has included TB contact investigation in its National TB Program Guidelines since at least 2010 [34]. Still, it was not until after the WHO 2013 endorsement of TB contact investigation that the program began actively implementing contact investigation [35]. Following this fundamental policy and practice shift, we adapted contact investigation to the Ugandan setting. Using a participatory, theory-informed approach, we co-developed a novel mHealth-facilitated implementation strategy with local partners. We then evaluated the adapted contact investigation strategy in a household-randomized, Type III hybrid implementation-effectiveness trial with nested mixed-methods studies of implementation outcomes [36]. While the trial found that home sputum collection and results reporting by text message did not increase the completion or yield of contact investigation, a rigorous mixed-methods evaluation plan provided a comprehensive explanation for the negative trial results in the mixed feasibility [37–39], low fidelity [40, 41], and substantial start-up and maintenance costs [42] of the adapted intervention and its implementation components. In previous case studies, we summarized our learnings from the implementation design phase [43] and the implementation of mHealth technologies phase [44]. In the current implementation science case study, we describe our insights from the study’s adaptation, implementation, and evaluation phases, highlighting the reasons behind our decisions and lessons learned from the outcomes.

## Methods

### Setting

Uganda is one of the world’s 30 high HIV-TB burden countries, with its annual TB incidence estimated at 200 per 100,000 [45] and its HIV prevalence at 5.4% [46]. Annual per capita GDP is USD 822 [47], placing Uganda at the upper end of the income band for low-income countries. The implementation setting for this study included seven public primary healthcare facilities in the capital city of Kampala. The Uganda Ministry of Health provides all TB diagnostic and treatment services in these facilities, including contact investigation, at no direct cost to patients. At the time of our study, standard contact investigation involved healthcare workers interviewing people diagnosed with TB (“index patients” or “persons with TB”) at healthcare facilities and then visiting their homes to interview their household contacts and screen them for TB. Household contacts were defined as anyone who had slept under the same roof as the person with TB for ≥ 1 day or night within the previous three months. Healthcare workers were trained to refer contacts with possible TB disease for TB evaluation at primary care clinics. Those defined as possibly having TB disease included all contacts under age 5; all contacts living with HIV/AIDS; and all contacts reporting one or more TB symptoms, including cough, fever, shaking chills, drenching night sweats, or weight loss (or failure to gain weight in children). At the clinic, healthcare workers were trained to perform a clinical assessment and refer contacts for microbiological evaluation of an expectorated sputum sample via GeneXpert MTB/RIF Ultra molecular testing, with smear microscopy as backup.

### Frameworks for implementation

**Fig. 1:**
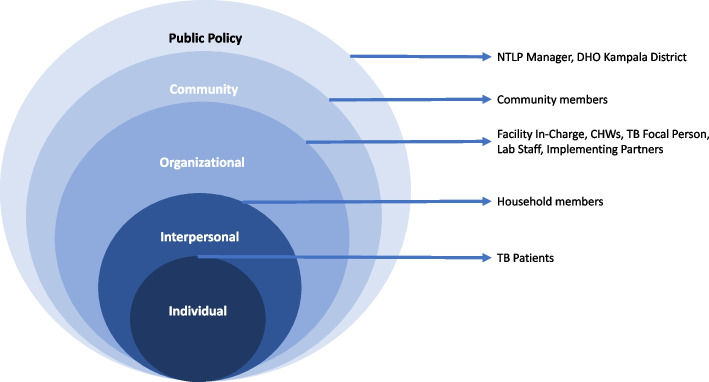
Socio-ecological model illustrating a multi-level partnership organized for adapting TB contact investigation and designing an implementation strategy. Legend: *NTLP*, National Tuberculosis and Leprosy Programme; *DHO*, District Health Officer; *TB*, tuberculosis; *CHWs*, Community Health Workers

We used two complementary frameworks to inform our adaptation of TB contact investigation and our design of the mHealth implementation strategy [48]. First, because we prioritized community engagement, we recruited implementation partners representing multiple levels of the social-ecological model (Fig. 1) [49].

We conducted focus group discussions with healthcare workers, community health workers, and contacts to elicit community preferences and suggestions about the three contact investigation activities [50]. Unfortunately, we inadvertently omitted one key group of partners, persons with TB, whom we subsequently found to be critical gatekeepers for reaching contacts [51]. Second, because we also placed a high priority on using theory to guide our implementation strategy, we applied the Capability, Opportunity, and Motivation influence Behavior (COM-B) model to classify the elicited barriers and facilitators and the linked Behavior Change Wheel (BCW) framework to identify potential adaptations and implementation components [50]. We selected the COM-B model and the BCW framework because they provide a comprehensive, systematic, and coherent approach to targeting individual and societal factors that influence individual health behaviors according to their underlying mechanisms of action [52]. Specifically, the BCW framework [50] offers nine intervention functions and seven policy categories that target one or more specific determinants of behavior within the COM-B model. We also used the Behavior Change Techniques Taxonomy, a list of 83 techniques previously used to change behaviors and overcome implementation barriers [53]. Having identified barriers, facilitators, intervention functions, and specific behavior change components, we reviewed these with our team of implementing partners and selected strategy components deemed person-centered and most appropriate to the local context [54].

### Intervention adaptation and delivery

We then conducted a quantitative process evaluation, using the TB contact investigation care cascade, to localize and prioritize specific gaps in intervention delivery [51]. We chose the cascade approach because it offered a familiar way for national TB program leaders and implementing partners to visualize the findings in our prior studies [55, 56] and in the HIV/AIDS response [57]. We identified low household visit rates and low TB and HIV evaluation completion rates as the largest gaps in the contact investigation care cascade. We also conducted qualitative studies with healthcare workers, community health workers (CHWs), and community members to elicit the most likely barriers to implementation, which included anticipated stigma for persons with TB and their contacts, high transportation costs for contacts, and long clinic wait times for contacts. To address these barriers, we adapted standard contact investigation to allow the enumeration of contacts, sputum collection for TB testing, and HIV testing at home, thereby minimizing the need for household contacts to attend clinics for screening and testing (Table 1). We also selected community CHWs to deliver home-based contact investigation because clinic and community informants reported that CHWs have greater freedom, flexibility, and perceived trustworthiness in the community than healthcare workers [50].

**Table 1:** Characteristics and components of the pre-implementation and context-adapted interventions and the tailored implementation strategy

**Characteristic**	**Standard of care intervention:**	**Context-adapted intervention:**	**Tailored implementation strategy:**
	**“Household TB contact investigation”**	**“Home-based TB contact investigation”**	**“mHealth facilitation”**
*Actors*	Clinic-based Health Care Workers	Community Health Workers	Mobile Health Tools
*Targets of action*	Persons with TB (to help reach contacts)	Persons with TB (to help reach contacts)	Community Health Workers
	Household contacts of persons with TB	Household contacts of persons with TB	Household contacts of persons with TB
*Actions*	1. Enumerate contacts at home	1. Enumerate contacts at home	1. Digital fingerprint scanning to track contacts
	2. Screen household contacts for TB at home	2. Screen household contacts for TB at home	2. Decision support to guide testing and referral
	3. Evaluate contacts for TB in *clinics*	3. Evaluate contacts for TB *at home*	3. *Automated-SMS reporting* of TB test results
		a. *Home-based* sputum collection	
		b. *Home-based* HIV-testing	
*Justification*	Not applicable	1. Reduce anticipated stigma for clients	1. Reduce contacts lost to follow-up
		2. Decongest clinic	2. Increase fidelity of screening and testing
		3. Minimize client costs	3. Improve delivery of results to clients
		a. Increase completion of TB testing	
		b. Increase completion of HIV testing	
*Temporality*	Timeline not specified	Household visit within one week of TB diagnosis	TB test results sent by SMS ≤ 3d of collection
*Dose*	One household visit	One household visit	One SMS message with TB test results
*Outcomes*		Number of contacts identified	Reach among eligible clients
		Number of contacts screened for TB	Feasibility, Acceptability, Appropriateness
		Number of contacts evaluated for TB	Cost
		Number of contacts tested for HIV	Fidelity
	Yield of TB diagnoses	Yield of TB diagnoses	Context

Column 1 lists the characteristics of a well-specified intervention/implementation strategy, as adapted from Proctor et al. [58]; Column 2 describes Household TB contact investigation as specified in Uganda in the pre-implementation phase; Column 3 describes Home-based TB contact investigation, the context-adapted form of the intervention; Column 4 describes mHealth Facilitation, the tailored implementation strategy

In the randomized trial, Actions 1 and 2 (including Actions 1 and 2 of the tailored implementation strategy) were delivered to all households, while Actions 3 (including 3a, 3b) were randomly allocated according to either the standard of care intervention or to the context-adapted intervention plus the tailored implementation strategy

*Abbreviations*: *SMS* Short messaging services, *TB* Tuberculosis

### Implementation strategy

Knowing that strategies consisting of multiple interacting components tend to be more effective than single-component ones [59], we proposed three elements to our mHealth strategy, each targeted to address a critical barrier to implementation (Table 1). First, the standard paper-based records for tracking and monitoring contacts may only partially capture all contact investigation activities, given the challenges of updating clinic registers with community-collected data and linking persons who move between clinics. Therefore, we proposed fingerprint scanning to help us accurately monitor patients and accurately link their results across multiple primary care clinics.

Second, TB screening, testing, and referral algorithms can be complex, given the need to consider each contact’s age, symptoms, and HIV/AIDS history, especially for CHWs, who have less education and training than regular healthcare workers. Therefore, we embedded an electronic decision-support system within our data collection system to guide CHWs in using TB screening, testing, and referral algorithms. We chose CommCare, a customizable, open-source survey application (Dimagi, Boston, MA) because it is designed explicitly for CHWs and is available on smartphones and feature phones.

Third, many contacts may not receive their TB evaluation results because returning to clinics is costly and frustrating with long clinic waits and tense interactions with healthcare workers. Thus, we used automated short messaging services (SMS) to report testing results to household contacts who had provided sputum at home. This element of the implementation strategy aimed to decrease overall losses to follow-up and increase the number of contacts initiating TB treatment after a positive test result.

### Design and measurement

We conducted a household-randomized trial comparing the implementation and effectiveness of standard, clinic-based TB contact investigation to the locally adapted intervention, home-based contact investigation, paired with the mHealth-facilitated implementation strategy (Table 1). We then used quantitative and qualitative methods to carry out a multi-stage evaluation of the implementation strategy, including its acceptability, appropriateness, feasibility, fidelity, and costs [60]. First, we conducted a detailed evaluation of delivery processes. Our careful design of the electronic case-record forms to capture each step made this possible. Next, we organized multiple focus group discussions with CHWs to better understand the adapted intervention’s acceptability, appropriateness, and feasibility and the mHealth implementation strategy. Third, we performed in-depth interviews with CHWs, persons with TB, household contacts, and facility administrators to help contextualize our findings on implementation outcomes. Fourth, we surveyed household contacts to further understand the fidelity of the implementation strategy, including how well it eliminated key barriers to completing TB contact investigation. Finally, we reviewed informal field reports to better understand how implementation fidelity evolved over time.

It can be challenging to synthesize findings from multiple data streams, a problem that implementation frameworks help solve. To understand the determinants of the fidelity of SMS and other mHealth tools [61], we chose the Consolidated Framework for Implementation Research (CFIR), which has been widely and successfully used to evaluate implementation, although less frequently in global health settings [62]. CFIR helped us identify several prominent barriers to and facilitators of mHealth implementation, including factors related to intervention characteristics, the inner setting, and the characteristics of individual implementers. Last, we again used the COM-B model to identify additional facilitators and barriers to HIV testing because it had proven usable, efficient, and easily interpretable during implementation planning.

### Ethics

All participants or their parents/legal guardians provided individual written informed consent for trial participation, with children 12–17 years also providing written assent. Those recruited to qualitative sub-studies gave separate verbal informed consent at the time of participation. Institutional review boards at Makerere University, the Uganda National Council for Science and Technology, and Yale University approved the study.

### Community engagement

We engaged key partners (Fig. 1) during the design and evaluation of the implementation strategy, including household contacts; CHWs; TB healthcare workers; and Uganda Ministry of Health staff overseeing the National Tuberculosis and Leprosy Programme on their perceptions of barriers to successfully implementing contact investigation [50]. We also interviewed household contacts about their mobile phone practices and preferences for receiving health information by phone [39]. During the analysis phase, we held dissemination sessions with CHWs, TB program officials, and other implementing partners to elicit their feedback on our conclusions and to ensure accurate inferences. We also presented our findings at local academic conferences to reach a broader array of Ugandan researchers and implementers.

## Results

From July 2016 to July 2017, we conducted a cluster-randomized controlled trial that recruited 919 household contacts from 372 households [36]. The adapted home-based intervention delivered with a tailored mHealth-facilitated implementation strategy performed similarly to standard clinic-based contact investigation. There were no significant differences between study arms for either the primary implementation outcome, completion of TB evaluation within 14 days (14% vs. 15%; risk difference − 1%, 95% CI − 9% to 7%, *p* = 0.81), or the primary effectiveness outcome, the yield of new TB cases among all contacts screened (1.5% vs. 1.1% odds ratio 1.34, 95% CI 0.42–4.24, *p* = 0.62) [36]. This section describes how we selected, collected, and interpreted implementation measures that ultimately helped explain the low implementation and effectiveness outcomes we observed in the trial. We also highlight learnings for the design and evaluation of implementation studies.

### Acceptability

We defined acceptability as the perception among participants and implementers that the intervention and implementation components were satisfactory. One challenge we identified in measuring acceptability, especially for mHealth technologies, is that acceptability can change as the actual or perceived usability of the intervention or implementation components evolves over time. Because technology evolves rapidly [33], the standard approach of measuring implementation outcomes only at project start and completion needs to be revised.

In this project, we used qualitative methods to evaluate the acceptability of the core (SMS) and peripheral (fingerprinting and electronic data collection) implementation components, as perceived by household contacts and CHWs. Informal interviews and field notes collected during the pilot phase suggested that the mHealth components were highly acceptable to household contacts and CHWs. Over time, however, several mHealth components’ acceptability decreased as hardware failures (e.g., cables, scanners, and tablets damaged by wear-and-tear) and software failures (e.g., compatibility problems arising after operating system updates) increased. Using quantitative short-survey instruments, such as the acceptability of implementation measure (AIM) [63] or the system usability scale (SUS) [64] to assess the implementation of these technologies might have allowed us to detect and correct such problems earlier. Additionally, household contacts found reporting of TB testing results less than acceptable as it did not permit direct and easy connection to CHWs (the toll-free, reply-only “HELP” function was insufficient) [38]. Even after receiving negative test results, contacts wanted a health worker to speak directly with them to confirm and explain the results, partly because of mistrust of the accuracy of results sent by SMS.

Reviews were also mixed for digital data entry using the customized CommCare application. Most CHWs initially reported excitement about the technology and expected it to simplify contact investigation. While the survey application, including the decision-support algorithm, remained popular during weekly supervisory meetings, other aspects became less acceptable over time. CHWs developed a mistrust of hardware and software components that were prone to malfunction or required a data connection [44, 65]. They also became impatient with the complex procedures required to register fingerprints or new telephone numbers in the system. End users reported rarely using and even avoiding frequently malfunctioning features, such as digital fingerprinting for patient identification. In contrast, they maintained a high level of enthusiasm for other core features of the survey application that consistently worked, such as the decision support tool.

### Appropriateness

We also evaluated the appropriateness of each intervention component, defined as its perceived fit or relevance in the new setting. As with the measure of acceptability, we recognized a need for short, reliable, and quantitative instruments for repeatedly measuring appropriateness over time to replace more subjective assessments such as field notes. Some components, like tablet-based data collection, were consistently perceived as appropriate throughout implementation. In contrast, other components, such as the wired connection between the digital fingerprint scanner and the tablet, were gradually perceived as less appropriate once newer, easier-to-use wireless Bluetooth connections became available locally.

In pilot phase surveys and focus groups and during the initial implementation of the mHealth strategy, household members perceived SMS as a highly appropriate medium for health communications in this setting, where SMS-ready feature phones were predominant [39]. However, over time, the appropriateness of SMS decreased as lower-cost messaging services with expanded features (e.g., WhatsApp) emerged and became popular with the target population. During the second half of the study, 33% of participants reported never checking SMS messages and overlooking critical information about TB test results when it was delivered [40]. Similarly, the hardware required to use the customized CommCare application was initially deemed appropriate for the setting. However, with new updates to software systems and newer technologies continually entering the market, the fit over time declined as the specific hardware (i.e., tablets and cables used for fingerprinting) could no longer be procured locally and instead had to be imported from abroad [44]. While it is difficult to predict how they will evolve, the need to adapt and update technologies should factor into decision-making and planning.

In contrast, home-based sputum collection and HIV testing, which were intervention adaptations rather than implementation strategy components, were perceived to be a good fit for the setting. CHWs reported that home-based TB and HIV testing remained popular with household contacts over time. By the end of the trial period, the national TB program had incorporated home-based sputum collection into its contact investigation guidelines, affirming its appropriateness in this setting [66].

### Feasibility

We defined feasibility as the extent to which a new strategy can be delivered successfully in a given setting. Existing tools for measuring feasibility were limited at the time of our trial, and a need for more standardized, quantitative measures of feasibility still exists. This need is especially relevant for mHealth components, where the required technical specifications and human resources may be challenging to forecast, especially if there are many potential end states. One potentially helpful approach that we employed was simulated stress testing to identify failure points, but this methodology may require substantial technical and programming expertise. Such approaches must also consider contextual risks, including power surges and failures, and the logistical barriers to obtaining replacement parts.

We evaluated the feasibility of intervention adaptations (home-based HIV testing and sputum collection) and the mHealth implementation strategy components (fingerprinting and SMS message delivery).

Home-based HIV testing was highly feasible in this setting [37], as a process evaluation showed that CHWs could deliver this service with high fidelity in 100% of consenting contacts and provide accurate results as measured by a rigorous external quality assurance program [37]. Both the CHWs and the clinic-based laboratory staff saw CHWs as capable of conducting HIV testing. In contrast, the feasibility of home sputum collection was mixed — CHWs collected sputum in just 37% of eligible contacts [41]. Still, when they were able to collect sputum, sample quality was similar to sputum collected in health facilities. Moreover, almost all samples were successfully transported and tested. Household contacts considered home-based sputum collection a convenient way to initiate evaluation for TB [41].

Finally, digital fingerprinting and SMS were highly infeasible because of the complexity of implementing such a system. Having only one expert in information technology (IT) on the local team led to a reliance on external specialists, making routine software maintenance, troubleshooting, and repairs more problematic. Future mHealth implementation programs should account for this gap and address it through IT workforce strengthening.

### Fidelity

We defined fidelity as the degree to which a strategy was implemented as intended [67].

Measuring the fidelity of mHealth components required greater customization than measuring the fidelity of other intervention components, such as HIV testing or sputum collection. The design of the software in some cases, and the design of the telephone network in others, prevented us from capturing relevant procedural metadata, such as SMS delivery confirmations or fingerprint scanning attempts. Nevertheless, we were able to add surveys and interviews to capture fidelity in other ways. Despite some reporting biases inherent in these methods, our multi-modal approach enabled us to determine the reasons for the observed implementation failures. Consequently, fidelity measures in mHealth studies may benefit from both generalizable measures of participant uptake and usage and more customizable measures of participants’ resulting health behaviors.

In our study, fidelity to the adapted evidence-based intervention and the mHealth strategy varied by component. CHW adherence to home-based HIV testing was high, but half of the eligible contacts declined to test. Adherence of CHWs and contacts to home-based sputum collection procedures was low-to-moderate. We found that the decision support elements of the mHealth strategy worked well. However, the text messaging lacked fidelity because of a coding error that led to 42% of SMS messages never being sent to household contacts. In addition, many participants did not open, read, or retain the content of the messages [40]. Finally, CHWs avoided using digital fingerprint scanning as they came to expect technical failures [65], limiting its overall utility as an identification tool.

### Implementation costs

We performed a formal cost analysis to better understand the scalability of our mHealth implementation strategy for home-based TB contact investigation [42]. We found that collecting and transporting sputum and conducting point-of-care HIV testing in the home had relatively low costs, given the availability of next-generation diagnostics to help facilitate this. More significant costs arose during the initial design and adaptation of the software used in the mHealth system. Expenses included the IT personnel necessary for the system’s design, testing, and maintenance and the continued monthly software costs. We also captured recurring hardware replacement costs as devices were lost, stolen, or broken. These expenses are frequently overlooked when modeling the cost-effectiveness of mHealth strategies. Overall, scaling up the strategy country-wide could help the Uganda Ministry of Health spread these high start-up costs across more users to make the strategy more affordable.

### Ethical considerations

Implementation research may give rise to particular ethical challenges that go beyond the focus of medical ethics and research ethics on individual protections and require implementers to apply the principles of public health ethics if an over-emphasis on individual protections threatens the science [68]. For example, our study sponsor required individual written informed consent from all participants, even though study activities posed no greater risk than encountered in routine practice. CHWs reported that obtaining consent lengthened the home visits considerably, reducing feasibility and changing the context in ways that, often undermined their ability to develop rapport with household members.

A second challenge related to implementation research ethics involves different perspectives on equipoise, the notion that when comparing two interventions in a trial, investigators should be genuinely uncertain about which is superior. Researchers and public health leaders felt that both the intervention and the control strategies were equally likely to achieve the desired outcomes because of the challenges of successfully delivering the complex intervention. In other words, they felt that contextual equipoise between arms was present. In contrast, CHWs saw the mHealth-facilitated strategy as superior, a perception that made at least one CHW uncomfortable delivering the standard strategy. Thus, on at least one occasion, a CHW offered home-based TB and HIV testing to household members assigned to the standard strategy because the CHW stated that it was her duty to provide what she perceived as the best available care. While we do not know how often this occurred, it is unlikely to have affected our results, given the low completion rates in both arms, unless the mHealth arm was truly inferior.

## Discussion

In this global implementation science case study, we describe our experiences developing, introducing, and evaluating a community-engaged, theory-informed implementation strategy to improve the delivery of household TB contact investigation in Uganda. Although the resulting trial failed to show improvements in the completion rate for TB evaluation among household contacts or in the yield of active TB diagnoses and treatments, we learned many valuable lessons for the TB program and implementation science. These lessons included learning how to work within diverse partnerships to develop a tailored implementation strategy for the delivery of TB contact investigation and recognizing that additional attention to the particular ethical challenges of implementation research is needed. Second, we demonstrated how to adapt a high-priority, evidence-based public health intervention to fit the local setting and how to work with public health partners to update national policies based on the results of an implementation trial. Third, our multi-disciplinary, multi-level evaluation plan identified the root causes of failed implementation. By identifying the intervention activities and implementation components that are less feasible, less acceptable, or less appropriate, we can prioritize the refinements needed to improve the fidelity of home sputum collection and results reporting and potentially reduce implementation costs. Finally, we identified the need for more accurate approaches to monitoring and evaluating the implementation of new technologies.

### Impact

While our overall implementation strategy was no more effective than standard contact investigation, we did identify the contextual factors that may be important to improve the acceptability, appropriateness, feasibility, and fidelity of future mHealth strategies. For example, the national TB program is piloting individual electronic data collection systems for contact investigation on the DHIS2 electronic data management platform. Similarly, our adaptations to contact investigation, including home HIV testing and home-based sputum collection, were incorporated into the updated Uganda national TB guidelines [66]. The translation of these results to policy occurred partly because these adaptations proved to be feasible and appropriate ways to make TB contact investigation more person-centered, which is a local and a global priority. We also found that tasking CHWs with contact investigation was feasible and freed overburdened healthcare workers to focus on clinic-based activities.

We also learned about challenges that can arise when randomization is used to test novel modes of service delivery in real-world settings. Specifically, we learned that assessments of equipoise may differ between individuals and over time. To avoid ethical concerns, including any perception of moral hazard among frontline implementers, we suggest assessing contextual equipoise formally in collaboration with a community advisory board rather than informally with health workers and program leaders, as we did in this study. In addition, to avoid contamination in implementation trials, we would discourage allocation procedures that require implementers to deliver intervention components to those in some randomization units and control strategies to others [69].

In addition, mHealth strategies may pose ethical challenges if they exacerbate health inequities. While most Ugandans reported owning or having access to a phone in our formative research [39], in practice, some participants were excluded, and others were unable to engage fully with the text messaging component because their access was unstable and limited by phone sharing, network instability, and lack of ready access to electricity for charging. As others have pointed out [70], requiring phones to access health interventions will tend to exclude the poorest in our societies, furthering inequities across wealth divides. While there are no simple, low-cost solutions for this, it must be at the forefront of researchers’ and implementers’ minds, as creating equitable and just solutions are the only way we will eliminate TB.

### Sustainability

Given the high demands on providers and high levels of associated burnout reported in LMICs [71], shifting appropriate tasks to CHWs offers an opportunity to enhance health-system capacity and service delivery [72]. Our adapted intervention and mHealth implementation strategy relied heavily on task-shifting of contact investigation procedures to CHWs, and they excelled at providing high-fidelity care to household contacts, who reported high levels of trust in CHWs. The major barrier for CHWs was inconsistent compensation and reimbursement, which systematic reviews have shown to be critical to the success of CHWs [73]. In addition, because CHWs cost less than traditional health workers and require less training, they may offer a more scalable and sustainable way of delivering contact investigation as long as quality-assurance mechanisms are in place.

On the other hand, our cost analyses suggested that the mHealth components of our implementation strategy are not affordable, even if they could be made more effective. One likely reason relates to the complexity of the system we designed and its reliance on novel platforms and technologies rather than on existing and widespread technologies within Uganda. In addition, given the need for more information technology experts in the local setting, lower-maintenance components are needed. Future mHealth initiatives should try to use software systems that can be sourced and maintained locally and accessible to all on ultra-low-cost devices.

## Conclusions

Overall, the low levels of implementation fidelity seen during the trial and the barriers we faced in accurately measuring implementation outcomes suggest that more work is needed to better plan, implement, and evaluate mHealth strategies, thereby improving the delivery of evidence-based interventions and health outcomes in the global health context. However, we were able to definitively demonstrate the value of implementation science methods in understanding what works and what does not work in designing, adapting, and evaluating implementation strategies in this context. This process may be particularly important for mHealth interventions, which have struggled to achieve continuity, integrate learnings from implementation failures in preliminary studies, and achieve successful implementation at scale.

## Data Availability

Data sharing does not apply to this article as no datasets were generated or analyzed during the current study.
